# Intra-abdominal Pressure Has a Good Predictive Power for 28-Day Mortality: A Prospective Observational Study Conducted in Critically Ill Children

**DOI:** 10.3389/fped.2020.567876

**Published:** 2020-10-20

**Authors:** Yujian Liang, Shaohua Tao, Bin Gu, Huimin Huang, Zhihai Zhong, Jingrong Shi, Xiangdong Guan, Wen Tang

**Affiliations:** ^1^Department of Pediatric Intensive Care Unit, The First Affiliated Hospital, Sun Yat-sen University, Guangzhou, China; ^2^Department of Pediatric Intensive Care Unit, Zhujiang Hospital, Southern Medical University, Guangzhou, China; ^3^Department of Critical Care Medicine, The First Affiliated Hospital, Sun Yat-sen University, Guangzhou, China; ^4^Department of Pediatric Surgery, The First Affiliated Hospital, Sun Yat-sen University, Guangzhou, China; ^5^Department of Data Mining and Analysis, Guangzhou Tianpeng Technology Co. Ltd., Guangzhou, China

**Keywords:** intra-abdominal pressure, normal values, in-hospital mortality, pediatric intensive care unit, predictor

## Abstract

**Objective:** Elevated intra-abdominal pressure (IAP) is associated with organ dysfunction in critically ill children. Thus far, the predictive value of IAP for mortality remains unknown. Moreover, only few studies determined normal IAP values in pediatric intensive care unit (PICU) children. This study aimed to determine the predictive value of IAP for mortality and calculate normal IAP values in PICU patients.

**Methods:** This prospective observational study was conducted in two PICUs of two tertiary care university teaching hospitals. Patients admitted to the PICU between December 2013 and November 2015 were included. IAP was determined by bladder pressure measurements performed every 8 h until 48 h or until PICU discharge. All patients (except neonatal patients) aged ≤ 14 years who were admitted to the PICUs and had no history of chemical neuromuscular blockade use, neurogenic bladder, or bladder surgery were enrolled. Binary logistic regression was used to analyze the predictive value of IAP for 28-day mortality. Receiver operating characteristic curves were generated to evaluate the prediction effect of IAP.

**Results:** Overall, 229 patients were enrolled. IAP (hazard ratio 1.09, 95% confidence interval [CI] 1.029–1.161, *P* = 0.004) and lactic acid (hazard ratio 3.04, 95% CI 1.769–5.21, *P* < 0.001) were independent predictors of 28-day mortality. Additionally, IAP had good predictive power for 28-day mortality, with an area under the curve of 0.74. The optimal cutoff point was 12.13 mmHg (sensitivity 0.58, specificity 0.80). The Youden index was 0.38.Furthermore, 111 (48.47%) patients without high-risk factors or clinical manifestations of IAH were analyzed to determine normal IAP values, which were 7.57 ± 2.85 mmHg (range, 1.98–13.16 mmHg). There were no significant differences in normal IAP values according to different diseases, sex, age, weight, or body mass index (BMI).

**Conclusions:** IAP has good predictive power for 28-day mortality. The optimal IAP cutoff point is 12.13 mmHg. The IAP reference range is 2.0–13.2 mmHg, which was not associated with factors such as sex, age, weight, and BMI in PICU children. We recommend that IAP be included in critical illness scoring systems in the future. IAP >12.13 mmHg may be more suitable for IAH definition in PICU patients.

## Introduction

Elevated intra-abdominal pressure (IAP) can lead to intra-abdominal hypertension (IAH), which is associated with dysfunction of the cerebrum and the digestive, respiratory, cardiovascular, and renal systems. Additionally, IAH has been reported as an independent risk factor for mortality in the pediatric intensive care unit (PICU) (1–6). Thus far, the predictive value of IAP for mortality in PICU patients has not been reported in the pediatric literature. Moreover, only few studies have examined the normal reference range of IAP in PICU patients (7, 8). According to the updated World Society of the Abdominal Compartment Syndrome (WSACS) guidelines (9), the normal IAP value in a critically ill child was 4–10 mmHg; IAH was defined as a sustained IAP elevation of >10 mmHg; the guidelines are based on data collected from a single-center study of children who were put on mechanical ventilation (MV) (7). However, a recent study identified MV as a risk factor for IAH; additionally, a positive end-expiratory pressure (PEEP) level of >10 cmH_2_O (1 cmH_2_O = 0.098 kPa) is considered a risk factor for IAH in children (10–13). In the PICU, not all children receive MV, and children not receiving MV are also at a risk of developing IAH. Therefore, it is unclear whether normal values of IAP in children not receiving MV are consistent with those in children receiving MV and whether such normal values of IAP defined by the WSACS guidelines are applicable to children without MV support. Notably, physiological variables such as old age and increased body mass index (BMI) have been reported as risk factors for IAH in adults (14–16). Only few studies have analyzed the influence of physiological variables on normal values of IAP in children (7, 8). The study conducted by Ejike et al. examined normal IAP values in different weight groups; however, they did not analyze the effects of other physiological parameters such as age, sex, or BMI. Therefore, it is unknown whether these physiological parameters affect the normal values of IAP in children.

Therefore, this prospective observational study aimed to determine the predictive value of IAP for 28-day mortality and calculate normal IAP values in PICU patients.

## Materials and Methods

### Study Design

This prospective observational study was conducted in two comprehensive PICUs of internal medicine and surgery (12-bed unit and 25-bed unit, respectively) in two tertiary care university teaching hospitals. A total of 437 children (156 and 281, respectively) admitted to the PICU between December 2013 and November 2015 were prospectively enrolled after obtaining written informed consent from the parent or guardian. The exclusion criteria were as follows: age ≤ 28 days (*n* = 82) or >14 years (*n* = 21), PICU admission for <24 h (*n* = 47), lack of parental consent (*n* = 6), use of chemical neuromuscular blockade (*n* = 15), and history of neurogenic bladder (*n* = 9) or bladder surgery (*n* = 28). Finally, 229 critically ill children were enrolled in the study, and the mortality predictive value was determined in these patients; 111 of the 229 patients did not have high-risk factors (Supplementary Table 1) (with the exception of physiological parameters) or clinical manifestations of IAH (9), and the normal IAP values were determined in these patients. The study protocol was approved by the Ethics Committees of the two study centers (2013, No. 170).

### Data Collection

The following parameters were recorded during admission: sex, age, weight, height, BMI, blood pressure, MV status, peak inspiratory pressure (PIP) and PEEP, use of an analgesic/sedative, blood gas analysis, main diagnosis, Pediatric Critical Illness Score (PCIS), Pediatric Risk of Mortality Score (PRISM III), Pediatric Logistic Organ Dysfunction (PELOD) score, and 28-day mortality. IAP was measured manually based on the bladder pressure measurements taken using a Foley catheter according to the standard procedure outlined in the guidelines (7). Briefly, the patient was placed in the supine position. The test was conducted when the patient was relaxed (sedated if necessary) to reduce interference with IAP values. Sterile saline was injected into the empty bladder (1 mL/kg, with a minimal installation volume of 3 mL and a maximum installation volume of 25 mL). The IAP was measured 30–60 s after the installation and at the end of expiration. The mid-axillary line at the iliac crest was considered as the reference. Finally, the level of the water column above the mid-axillary line at the end of expiration reflected the IAP value. The value was expressed in cmH_2_O for manual measurements, and hence, the value needed conversion to mmHg (1 cmH_2_O = 0.73 mmHg). The IAP was measured every 8 h until 48 h or until the patient was discharged from the PICU. We selected the IAP mean value during the observation period to determine normal IAP values and assess the predictive value of IAP for mortality.

### Statistical Analysis

Statistical analyses were performed using IBM SPSS version 22.0 (IBM, Armonk, NY, USA) and R 3.6.2. Descriptive analyses were performed, and the values were expressed as mean ± standard deviation for normally distributed variables, median [interquartile range (IQR)] for non-normally distributed variables, and frequency (proportions, %) for categorical variables. Data were analyzed by stratifying the patients based on sex (male or female), age (>28 days−1 year, >1–7 years, and ≥7 years), weight (≤ 10 kg, >10–20 kg, and ≥20 kg), and BMI [categorized into three groups according to the percentile curve (17): ≥P85, overweight; P5–85, normal; and < P5, underweight]. Differences in continuous variables between the two outcome groups were compared using the independent samples *t*-test or Mann-Whitney U test. Differences in categorical variables were assessed using the chi-square test or Fisher exact test if indicated. IAP among different diseases were tested using analysis of variance and *post-hoc* multiple comparisons were tested using a Bonferroni adjustment.

We calculated the predictive value of IAP for 28-day mortality using binary logistic regression. Given the restriction of death event cases, we included only the highly correlated possible predictors, i.e., IAP, PaO_2_/FiO_2_, lactic acid levels, and MV, in the multivariable models to ensure sufficient statistical power. The Hosmer-Leme show goodness-of-fit test, Akaike information criterion (AIC), and Bayesian information criterion (BIC) were used to assess how well the model fitted the data. Recall, precision, F1 score, and area under the curve (AUC) of the receiver operating characteristic (ROC) curve were determined to evaluate the prediction effect of the models. The optimal cutoff value of IAP for predicting 28-day mortality was determined using time-dependent ROC curve analysis (survival ROC analysis). All statistical analyses were performed with a significance level of 0.05.

## Results

### Predictive Value of IAP for 28-Day Mortality in the PICU

A total of 229 children were enrolled in the study, and they underwent regular IAP monitoring. Demographic characteristics were compared between survivors and non-survivors using the binary classification model (Table 1). Binary logistic regression showed that IAP (*P* = 0.004) and lactic acid (*P* < 0.001) were independent predictors of 28-day mortality (Table 2). The area under the ROC curve (AUC), recall, and F1 score were 0.818, 0.911, and 0.511, respectively. Furthermore, the 1.09-fold risk of mortality increased with a 1-mmHg higher IAP (hazard ratio 1.09, 95% CI1.029–1.161, *P* = 0.004) and the 3.04-fold risk of mortality increased with a 1 mmol/L higher lactic acid (hazard ratio 3.04, 95% CI1.769–5.21, *P* < 0.001).

**Table 1:** Demographic characteristics of survivors and non-survivors.

	**All (*n* = 229)**	**Survivors (*n* = 198)**	**Non-survivors (*n* = 31)**	***P***
Age, y	2.0 (1.00–6.00)	2.55 (1.00–6.00)	2.0 (0.96–8.50)	0.91
Boys	153 (66.81)	135 (68.18)	18 (58.06)	0.27
Weight (kg)	13.6 (9.50–20.00)	13.75 (9.50–20.00)	13.0 (9.65–23.05)	0.94
Height (cm)	92.0 (75.00–113.20)	92.0 (75.25–113.00)	88.0 (72.00–134.00)	0.91
BMI (kg/m*^2^*)	15.55 (14.03–17.01)	15.53 (14.04–16.92)	15.62 (13.90–18.27)	0.53
IAP (mmHg)	8.82 (6.98–12.49)	8.45 (6.62–11.67)	13.23 (9.38–18.19)	<0.001
MAP (mmHg)	60.0 (52.70–75.30)	58.3 (55.00–75.00)	64.0 (50.00–76.50)	0.99
APP (mmHg)	49.7 (42.50–63.80)	49.6 (43.20–63.77)	50.5 (36.05–63.55)	0.26
PCO_2_ (mmHg)	45.0 (40.00–45.00)	45.0 (42.00–45.00)	41.0 (35.00–50.00)	0.41
P/F (mmHg)	300.0 (280.00–300.00)	300.0 (300.00–300.00)	290.0 (218.00–300.00)	0.04
MV, n(%)	82 (35.81)	61 (30.81)	21 (67.74)	<0.001
PIP (cmH_2_O)	20.0 (20.00–22.00)	20.0 (20.00–21.00)	20.0 (20.00–22.00)	0.17
PEEP (cmH_2_O)	4.0 (4.00–5.00)	4.0 (4.00–5.00)	4.0 (4.00–5.25)	0.15
Lactic acid (mmol/L)	29 (12.66)	13 (6.57)	16 (51.61)	<0.001
Sedation/Analgesia, n (%)	85 (37.12)	77 (38.89)	8 (25.81)	0.16
Organ dysfunction	0.0 (0.00–0.00)	0.0 (0.00–0.00)	0.0 (0.00–0.50)	0.73
PELOD	0.0 (0.00–2.00)	0.0 (0.00–2.00)	0.0 (0.00–2.00)	0.59
PRISMIII	12.0 (10.00–16.00)	12.0 (10.00–16.00)	11.0 (10.00–18.00)	0.95
PCIS	92.0 (84.00–100.00)	92.0 (84.00–100.00)	92.0 (82.00–100.00)	0.86

*BMI, body mass index; IAP, intra-abdominal pressure; MAP, mean arterial pressure; APP, abdominal perfusion pressure (APP = MAP–IAP); P/F, PaO_2_/FiO_2_; MV, mechanical ventilation; PIP, peak inspiratory pressures; PEEP, positive end-expiratory pressure; PRISM III, pediatric risk of mortality score; PELOD, pediatric logistic organ dysfunction score; PCIS, pediatric critical illness score*.

**Table 2:** Coefficient of the binary logistic regression model to access the predictive value of intra-abdominal pressure for 28-day mortality.

**Variable**	**β**	**Standard error**	**Wald**	**df**	**P value**	**OR (95% CI)**
Intercept	−5.107	0.959	28.383	1	<0.001	0.006 (0.001–0.04)
IAP	0.089	0.031	8.365	1	0.004	1.093 (1.029–1.161)
MV	0.607	0.544	1.244	1	0.265	1.834 (0.632–5.327)
Lactic acid	1.111	0.276	16.25	1	<0.001	3.036 (1.769–5.21)
P/F	0.001	0.002	0.153	1	0.696	1.001 (0.997–1.004)

IAP, intra-abdominal pressure; MV, mechanical ventilation; P/F, PaO_2_/FiO_2_.

*Hosmer-Lemeshow goodness-of-fit = 5.677; P = 0.683; Akaike information criterion (AIC) = 144.322, Bayesian information criterion (BIC) = 161.491; Area under Curve (AUC) = 0.818, Recall = 0.911, F1 = 0.511*.

**Figure 1:**
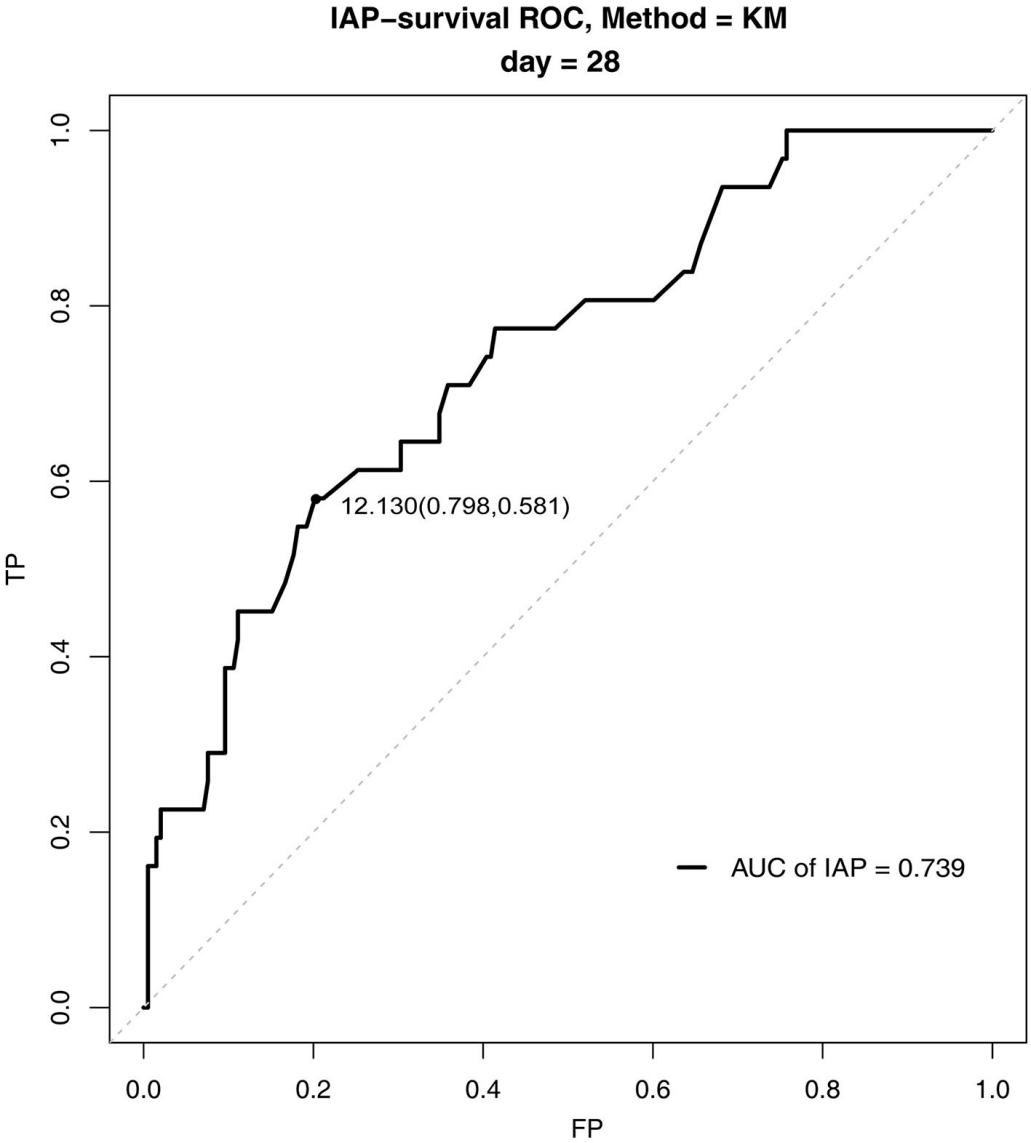
Survival receiver operating characteristic curves for intra-abdominal pressure for 28-day mortality in the pediatric intensive care unit.

The survival ROC curve analysis revealed that the IAP had good predictive power for 28-day mortality (Figure 1), with an AUC of 0.74 (SD 0.002, 95% confidence interval 0.73–0.74). The optimal cutoff point was 12.13 mmHg (sensitivity 0.58, specificity 0.80), and the Youden index was 0.38.

### Assessment of Normal IAP Values in PICU Patients

**Figure 2:**
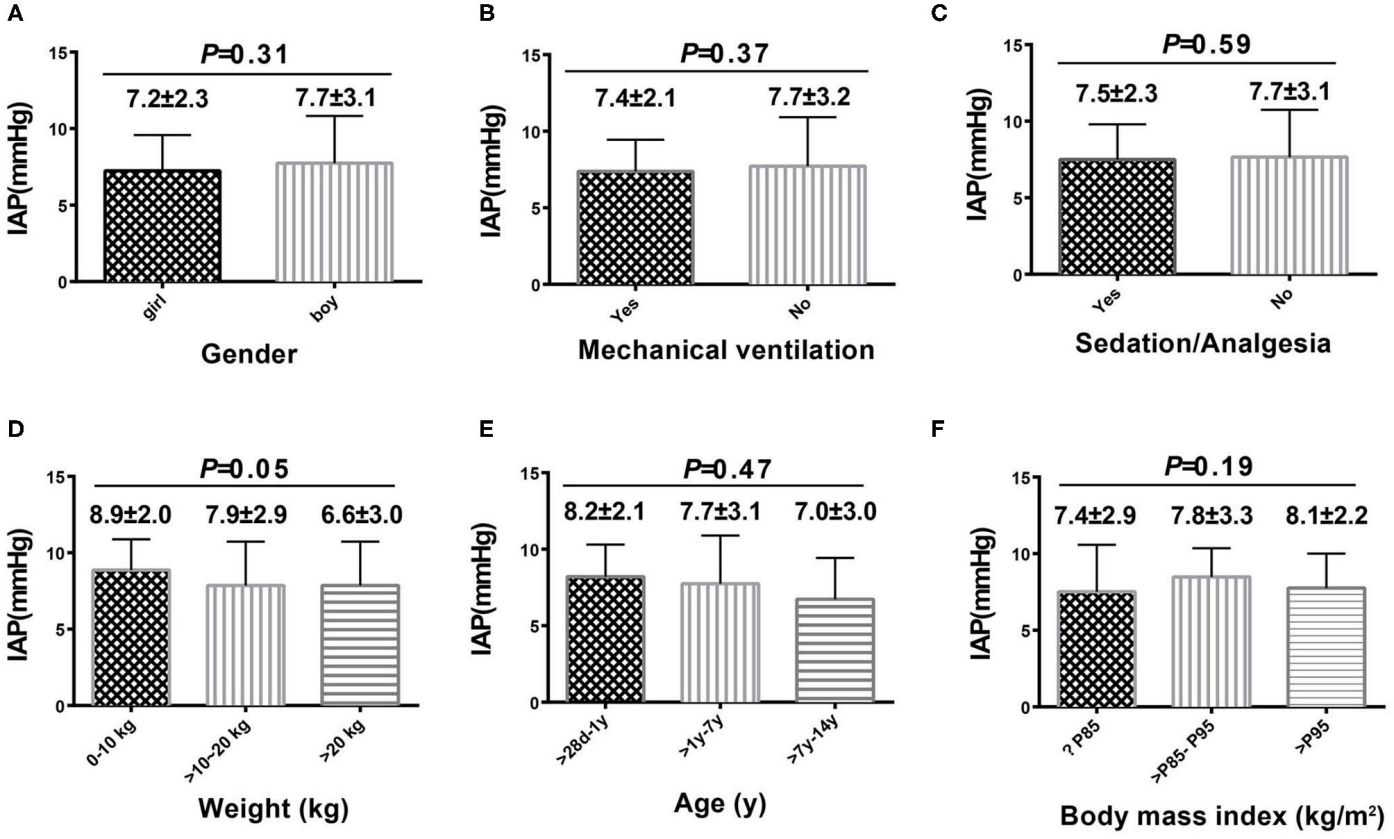
Intra-abdominal pressure according to sex, age, weight, body mass index, use of mechanical ventilation, and use of sedatives or analgesics. Comparison among each subgroup was shown in **(A–F)**.

A total of 111 patients without high-risk factors or clinical manifestations of IAH were analyzed to determine normal IAP values. There were no significant differences in IAP values based on the presence of different diseases (Table 3). The mean (±SD) normal IAP value was 7.57 ± 2.85 mmHg (range, 1.98–13.16 mmHg). Median patient age was 3 (IQR 1.42–6) years. The mean (±SD) values of weight and BMI were 17.31 ± 9.58 kg and 15.94 ± 2.74 kg/m^2^, respectively. There were no significant differences in IAP based on different sex, age, weight, and BMI (Figure 2). During the study period, 32.4% (*n* = 36) of patients received MV. The PEEP value was 4.5 ± 0.5 cmH_2_O in patients who received MV. There was no significant difference in IAP between the MV and non-MV groups (*P* > 0.05).

**Table 3:** Normal intra-abdominal pressure values in different diseases.

**Category of diseases**	**Ratio**	**IAP, mmHg**	***t***	***P***	***F***	***P***
Infection of the central nervous system	(33/111) 29.7%	6.7 ± 2.0	1.59	0.04	1.40	0.21
Diseases of the respiratory system	(52/111) 46.8%	7.6 ± 1.9	−0.14	0.86		
Non-abdominal trauma diseases	(3/111) 2.7%	6.6 ± 1.0	0.59	0.07		
Neck and oral surgery	(13/111) 11.7%	5.7 ± 3.4	1.48	0.18		
Skin surgery (non-abdominal wall)	(3/111) 2.7%	6.4 ± 1.8	0.57	0.24		
Hematologic diseases	(7/111) 6.3%	10.3 ± 3.2	−1.97	0.04		

*IAP: intra-abdominal pressure*.

## Discussion

In this study, we determined the predictive value of IAP for 28-day mortality in PICU patients. Moreover, we assessed the normal reference range of IAP in PICU patients and the influence of physiological variables on IAP. We analyzed the possible predictors of 28-day mortality among all children undergoing IAP monitoring during the study period, and we found that IAP and lactic acid were the independent predictors of 28-day mortality. The survival ROC curve analysis determined that IAP had a good predictive power for 28-day mortality. Thus, we recommend that IAP should be included in critical illness scoring systems in the future. In addition, we also analyzed normal IAP values in PICU children and found that there were no significant differences in IAP values based on disease types. Our findings suggest that the same standard for normal IAP values can be applied to patients with different types of diseases. Finally, the normal reference range of IAP was 1.98–13.16 mmHg. The optimal cutoff point for the predictive power of 28-day mortality was 12.13 mmHg. Thus, we recommend that IAP > 12.13 mmHg might be more suitable than IAP > 10 mmHg for IAH definition in children.

The study is novel because, to the best of our knowledge, no studies, to date, have reported the predictive value of IAP for 28-day mortality in PICU patients and only a few studies have examined normal IAP values in PICU patients or have analyzed the influence of physiological variables on normal IAP values in children. This is the first study to identify that, in addition to lactic acid, IAP is an important predictor for mortality in the PICU. In previous studies, lactic acid levels have been confirmed as a very important risk factor for 28-day mortality (18, 19). IAP is an important mortality predictor, possibly because IAP can affect many organ functions both inside or outside the abdominal area. Additionally, persistently increasing IAP can also lead to the development of multiple organ dysfunction syndromes (1–6); hence, IAP may have a very important effect on 28-day mortality. None of the previous scoring systems on the severity of illness have included IAP as an indicator in their analyses (20, 21). We recommend that IAP, which was verified as an important predictor of 28-day mortality in this study, be included in critical illness scoring systems in the future.

In view of the importance of IAP for PICU patients, the other objective of this study was to assess the normal reference range of IAP in PICU children. It is well-known that children's development is a dynamic process; thus, normal IAP values may differ in different age groups. Old age was found to be an independent risk factor of IAH in adult patients admitted to the intensive care unit (7, 13). The IAP level is related to abdominal wall compliance, and the high incidence of IAH in elderly patients is related to poor abdominal wall compliance (22). On the contrary, our results exhibited a trend of higher IAP values in younger patients and those with a lower weight. However, these findings were not significant. Ejike et al. (8) found that the normal IAP values were similar between different weight groups among critically ill children; this may be due to the immature development of the abdominal wall in children. There may be few differences in terms of compliance of the abdominal wall among different age groups.

In contrast to studies in adults, we did not find any relationship between IAP and BMI in the different BMI percentile groups. Lambert et al. (23) found a correlation between IAP and the sagittal abdominal diameter, which is an index of the degree of central obesity. Obesity or increased BMI is a risk factor for IAH in adults (14, 15, 24, 25). However, our study found no significant differences in IAP between children with and without obesity. These findings are similar to those obtained by Ejike et al. (26) and Horoz et al. (27), who reported no relationship between IAH and BMI. The different effects of BMI on IAP might be due to differences in the distribution of body fat and the muscle composition of the abdominal wall between adults and children.

MV is considered to be a risk factor for IAH (24, 28). In contrast to previous studies, we did not find any significant difference in IAP between patients with and without MV. This may be associated with low PEEP levels (4.5 ± 0.5 cmH_2_O) found in our MV patient group. The 2013 WSACS guidelines state that PEEP levels >10 cmH_2_O are regarded as one of the risk factors for IAH (7). However, Verzilli et al. (29) found that the reduction in splanchnic blood flow is limited at PEEP levels <10 cmH_2_O and is more pronounced at elevated PEEP levels ranging between 15 and 20 cmH_2_O. Our study reconfirmed these findings. As mentioned, Ejike et al. (8) did not report the PEEP levels of patients in their study on normal IAP values; thus, it is uncertain whether the PEEP values in their study population were similar to those observed in ours'. Our study indicated that a low PEEP level during MV may have little effect on IAP. Nevertheless, this relationship needs to be clarified in future studies.

Our study results showed that the mean (±SD) normal IAP value was 7.57 ± 2.85 mmHg (range, 1.98–13.16 mmHg) for PICU patients. This value is similar to the data reported by Ejike et al. (7 ± 3 mmHg); the latest guidelines define IAH mainly based on Ejike et al.'s data (8) and IAH was defined as an IAP value of >1SD (IAP > 10 mmHg) (7). Based on the optimal cutoff point of 12.13 mmHg for 28-day mortality prediction, we recommend that it may be better to define IAH as IAP >12.13 mmHg because it is a better predictor of death. In addition, increasing the threshold for IAH diagnosis might reduce unnecessary medical intervention, but further studies are needed to confirm its effect on prognosis.

This study had some limitations. First, this was a prospective observational study conducted in two university-affiliated hospitals. Therefore, selection bias may have been present, especially in the MV subgroup analysis. Second, only one-third of the patients were on mechanical ventilator support; when considering all patients, disease severity scores were not high; therefore, generalization of the results to all critically ill patients is not possible. Hence, future studies with large populations are needed. Third, the PEEP settings of the patients included in the study were generally low; thus, it is difficult to draw definitive conclusions regarding the effects of MV on IAP. Nevertheless, it can be inferred that when PEEP is ≤ 5 cmH_2_O, the effect of MV on IAP PEEP may not be significant. Fourth, based on the optimal cutoff point of 12.13 mmHg for 28-day mortality prediction, we recommended that it may be better to define IAH as IAP >12 mmHg. Whether increasing the threshold would affect prognosis requires further study with long-term follow-up.

In conclusion, IAP had good predictive power for 28-day mortality, and the optimal IAP cutoff point was 12.13 mmHg. We recommend that IAP be included in critical illness scoring systems in the future. The reference range for IAP was 2.0–13.2 mmHg, which did not show any association with physiological factors such as sex, age, weight, and BMI in PICU children. IAP >12.13 mm Hg may be more suitable for IAH definition in PICU patients.

## Data Availability Statement

The raw data supporting the conclusions of this article will be made available by the authors, without undue reservation.

## Ethics Statement

The studies involving human participants were reviewed and approved by The First Affiliated Hospital, Sun Yat-sen University. Written informed consent to participate in this study was provided by the participants' legal guardian/next of kin.

## Author Contributions

YL and ST designed the study, participated in data collection and interpretation, and drafted the manuscript. BG was involved in data collection. HH and ZZ performed the initial data analyses. JS revised the database and performed the statistical analysis. XG and WT participated in datainterpretation and critically revised the manuscript. All authors read and approved the final manuscript.

## Conflict of Interest

JS employed as a data mining analyst in Guangzhou Tianpeng Technology Co., Ltd. The remaining authors declare that the research was conducted in the absence of any commercial or financial relationships that could be construed as a potential conflict of interest.

## Abbreviations

AUC: area under the curve
BMI: body mass index
IAH: intra-abdominal hypertension
IAP: intra-abdominal pressure
MV: mechanical ventilation
OI: oxygen index
PCIS: pediatric critical illness score
PEEP: positive end-expiratory pressure
PELOD: pediatric logistic organ dysfunction
PICU: pediatric intensive care unit
ROC: receiver operating characteristic
SD: standard deviation
WSACS: world society of the abdominal compartment syndrome.
