# Supplementary material for: Intra-abdominal Pressure Has a Good Predictive Power for 28-Day Mortality: A Prospective Observational Study Conducted in Critically Ill Children
Source: Front Pediatr. 2020 Oct 20;8:567876. doi: 10.3389/fped.2020.567876 (PMC7606408; doi:10.3389/fped.2020.567876)
Supplement: Supplementary file 1 [file Table_1.DOCX]

**Table S1. Risk factors and clinical evidence for intra-abdominal hypertension according to 2013 guidelines.**

| **Category** | **Risk factors** |
| --- | --- |
| **Diminished abdominal wall compliance** | Abdominal surgery  Major trauma  Major burns  Prone positioning |
| **Increased intra-abdominal contents** | Acute pancreatitis  Distended abdomen  Hemoperitoneum/pneumoperitoneum Intra-peritoneal fluid collections  Intra-abdominal infection/abscess  Intra-abdominal or retroperitoneal tumours  Laparoscopy with excessive insufflations pressures  Liver dysfunction/cirrhosis with ascites  Peritoneal dialysis |
| **Increased intra-luminal contents** | Gastroparesis/gastric distention/ileus  Colonic pseudo-obstruction  Volvulus |
| **Capillary leak/fluid resuscitation** | Acidosis  Damage control laparotomy  Hypothermia  Massive fluid resuscitation or positive fluid balance  Polytransfusion |
| **Others/miscellaneous** | Bacteraemia  Coagulopathy  Increased head of bed angle  Massive incisional hernia repair  Mechanical ventilation  Obesity or increased body mass index  Positive end expiratory pressure >10 cmH_2_O  Peritonitis  Pneumonia  Sepsis  shock or hypotension |
